# Immunotherapy for Colorectal Cancer

**DOI:** 10.3390/cancers9050050

**Published:** 2017-05-11

**Authors:** Patrick M. Boland, Wen Wee Ma

**Affiliations:** 1 Roswell Park Cancer Institute, Department of Medicine, Elm and Carlton St, Buffalo, NY 14263, USA; Patrick.Boland@RoswellPark.org; 2 Mayo Clinic, Division of Medical Oncology, 200 First St. SW, Rochester, MN 55905, USA

**Keywords:** colon cancer, immunotherapy, anti-PD1

## Abstract

The recent success of anti-PD1 drugs in metastatic colorectal cancer patients with mismatch repair deficiency generated overwhelming enthusiasm for immunotherapy in the disease. However, patients with mismatch repair deficient colorectal cancer represent only a small subset of the metastatic population. Current research focuses on advancing immunotherapy to earlier stages of the disease including adjuvant and first-line metastatic settings, and on inducing sensitivity to immune checkpoint inhibitor therapy through a combinatorial approach. Here, we review the contemporary understanding of the immune and molecular landscape in colorectal cancer and discuss ongoing clinical trials evaluating novel combination regimens based on immune checkpoint inhibitors.

## 1. Introduction

Treatments focused on altering the immune system have recently made their way broadly into clinical oncology practice, based upon the successes seen with immune checkpoint inhibitors. While multiple cancers of the digestive tract have seen preliminary evidence of efficacy, colorectal cancers remain the steadfast exception. By and large, colorectal cancer has not benefited from immunotherapeutics; however, emerging data demonstrates that subsets of patients, those with hypermutated colorectal cancers, may benefit from immune checkpoint inhibitors. In addition, combinatorial approaches are evolving which may ultimately overcome this relative resistance across colorectal cancers. This article will provide an overview of the molecular and immunologic landscape, as well as a survey of immunotherapeutics currently under clinical evaluation in colorectal cancer.

## 2. Colorectal Cancer: Molecular and Immunologic Landscape

### 2.1. Molecular Alterations in Colorectal Cancer

In part due to the presence of clear precursor lesions, the step-wise pathogenesis of colorectal cancer was well described over two decades ago [1]. The efforts of the Cancer Genome Atlas (TCGA) and international consensus groups have collectively taken important steps to form consensus definitions of the colorectal cancer subtypes, with the aim of aiding future research efforts [2]. The majority of colorectal cancers demonstrate activation of the wnt/B-catenin pathway, in part due to inactivation of the tumor suppressor gene, *APC*. Relevant to therapeutic targeting, in metastatic disease RAS (KRAS or NRAS), mutations are seen in over 50% of patients, with BRAF mutations seen in 5–10% [3,4]. Additional emerging targets include HER-2 amplifications, seen in 2–5% of all colorectal cancers [5]. When considering genomic instability across various cancer types, colorectal cancers fall in the middle of the pack in terms of the average tumoral mutation load, though there is marked heterogeneity [6].

A subset of colorectal cancers possesses markedly elevated mutational rates. Predominantly, these tumors are characterized by dysfunction of the mismatch repair genes (microsatellite high or MSI-H). MSI-H tumors make up a minority of colorectal cancers, with decreasing frequency in more advanced stage disease. The prevalence of MSI-H in stage II, III and IV colorectal cancers stands at 22%, 12%, and 3%, respectively [7,8]. A small fraction of hyper-mutated tumors possesses polymerase mutations, specifically within the catalytic domain of DNA polymerase epsilon (POLE) or delta (POLD1). These hypermutated tumors are of great relevance in our current understanding of colorectal cancer subtyping and the role of immunotherapy.

### 2.2. Colorectal Cancer Subtypes

Four consensus molecular subtypes (CMS) of colorectal cancer have recently been agreed upon in a unification of prior classification criteria [9]. This classification system is based upon gene expression assays, similar to the determination of consensus breast cancer subtypes. At present, this is predominantly a classification with application to research rather than routine patient care. Interestingly, recent data has suggested that these subtypes may be accurately assigned through straightforward IHC based assays, though this remains to be validated in additional data sets [10]. CMS 1 tumors (MSI Immune, 14%) are characterized by hypermutation, MSI, and strong immune activation. CMS2 (Canonical, 37%) are epithelial, with chromosomal instability (CIN) and prominent WNT and MYC signaling activation. CMS3 (Metabolic, 13%) are epithelial, characterized by metabolic dysregulation. Finally, CMS4 (Mesenchymal, 23%) possesses prominent transforming growth factor β (TGF-β) activation, stromal invasion and angiogenesis. A remaining 13% possess mixed features.

A recent analysis examined several independent cohorts of colorectal cancers, with the goal of better describing the tumor microenvironment as it pertains to the CMS subtypes [11]. While CMS1 tumors are characterized by overexpression of genes specific to cytotoxic lymphocytes, CMS2 and CMS3 tumors demonstrate low inflammatory and immune signatures. On the other hand, the CMS4 subtype expresses markers of lymphocytic and monocytic origin and is characterized by an angiogenic, inflammatory and immunosuppressive signature, with a high density of fibroblasts seen on histologic examination. Thus, different strategies may be required for the success of immunotherapy in the various tumor subtypes. Immune checkpoint inhibition and therapies which might reactivate a stunted immune response may have greatest success in CMS1 tumors. On the other hand, CMS4 tumors will more likely require an approach which targets the suppressive monocytoid cells and related cytokines, alone or in combination with immune checkpoint inhibition. CMS2 and CMS3 tumors represent the classic ‘cold’ tumors, which might benefit from an immunogenic stimulus, such as radiation, a vaccine, or a co-stimulatory compound as a major part of the strategy. These are all strategies in development at present.

### 2.3. Microsatellite Instability

As a major component of the CMS1 category of colorectal cancers, MSI-H colorectal cancers deserve individual attention, as they represent the initial subset of colorectal cancers where immunotherapies have seen success. Microsatellite instability (MSI) is a marker of dysfunctional mismatch repair proteins within a tumor; MSI was first utilized on a clinical level to identify patients who should undergo germline testing for Lynch Syndrome [12]. However, it is important to understand that while 15% of colorectal cancers are noted to be MSI-H, only approximately 3% of all colorectal cancer possess a germline MMR mutation (Lynch Syndrome) [13,14]. Thus, the majority of MSI-H colorectal cancers are sporadic, due to acquired somatic defects in MMR gene function, most commonly secondary to hypermethylation of the MLH1 promoter [15]. Less commonly, two separate somatic mutations can induce mismatch repair deficiency [16].

MSI is a PCR based assay wherein typically five microsatellites are evaluated for instability; if ≥2/5 are unstable, the sample is deemed to be MSI-H. Alternatives results are MSI-L (1/5) or MSS (0/5), with MSI-L and MSS being equivalent for the purposes of this discussion. An alternative assay, which is a similarly sensitive screening test for Lynch Syndrome involves assessment of the MMR proteins by IHC: MLH1, MSH2, MSH6, and PMS2. An absence of staining or a ‘negative’ result is equivalent to an MSI-H tumor; a tumor that is negative for one or two MMR proteins is referred to as mismatch repair deficient (dMMR). The MMR assay has some potential advantages over the true MSI assay, as this can be performed in the vast majority of pathology laboratories, is considerably less expensive, and can allow for more focused mutational testing when considering Lynch Syndrome work-up.

Relevant to the present discussion, MSI-H tumors have long been recognized to carry a better prognosis in early stage colorectal cancer [17]. While such tumors are associated with BRAF mutations, synonymous with medullary histology, more commonly poorly differentiated and mucinous, MSI-H tumors are also more frequently characterized by a robust, “Crohn’s-like” lymphocytic infiltrate [18]. This may account for the improved prognosis in early stage disease. The robust lymphocytic response was previously postulated to be related to an immunogenic, high neo-antigen burden, due to the intrinsically elevated mutational rate. In-depth genomic analysis recently supported this concept [19]. However, that the immune system could be therapeutically targeted in colon cancer, specifically MSI-H colon cancer, was not universally accepted until long after the first report of such an event. As part of the initial clinical investigation of nivolumab (Opdivo), amongst the many colorectal cancer patients who derived absolutely no benefit, one patient achieved a complete response which was durable off therapy for over 3 years [20]. Diligent investigation demonstrated the patient to have an MSI-H tumor, with infiltrating macrophages and lymphocytes which were PD-L1+. This finding further promoted the hypothesis that perhaps these inflamed tumors could be targeted with developing immunomodulatory drugs: PD-1 inhibitors.

### 2.4. DNA Polymerase Mutations

The TCGA data in 2013 highlighted that a sizeable proportion of hypermutated tumors are not MSI-H: 7/30 (23%) samples tested. Rather, the majority of these hypermutated, MSS tumors are characterized by mutations in POLE [2]. Mutations in POLE and POLD have been identified in kindred affected by colorectal cancer or polyposis, confirmed in multiple cohorts [21,22]. Mutations in these critical genes map to the proof reading domains of DNA polymerases ε and δ, impairing the correction of mispaired bases and markedly affecting the fidelity of DNA replication [21,23]. As such, the tumors appear to have an “ultramutator” phenotype. The true frequency of clinically significant polymerase mutations is unclear and most probably varies by stage and patient characteristics, though in one report, of 224 tumors undergoing analysis, three (1.3%) were identified to have a POLE mutation [23]. Disparate results have been seen in other analyses. In a cohort of patients with polyposis or who fulfilled Amsterdam criteria, POLE mutations were noted in 1.5%, whereas a population-based German cohort reported a considerably higher rate of mutations via classic Sanger Sequencing (12.3%) [24,25]. Importantly, there are clinical anecdotes of responsiveness to PD-1 inhibition in POLE mutant endometrial cancer and more recently in POLE mutant colorectal cancer [26,27]. In sum, while POLE mutations seem uncommon, such mutations may well prove important as the role of immune checkpoint inhibitors evolves in colorectal cancer.

## 3. Key Immunotherapeutic Trials in Colorectal Cancer

An early study utilizing a CTLA-4 antagonist mAb, Tremelimumab, demonstrated the possibility of activity of immune checkpoint inhibitors in colorectal cancer, producing one response durable to 6 months [28]. However, there was only one case amongst the 47 patients treated which did not provoke enthusiasm to support further investigation. When PD-1 inhibitors first made a splash in the clinic, there were signs of durable response and activity in multiple different tumor types, including the MSI-H case previously described [29]. Below, initial reports and ongoing investigations of immune checkpoint inhibitors in colorectal cancer are detailed. Table 1 summarizes results of several key investigations to date.

### 3.1. Microsatellite Unstable (MSI-H) Trial Data

Based upon knowledge of the immunogenic tumor microenvironment in MSI-H tumors, as well as the documented dramatic response in such a patient with anti-PD-1 therapy, a trial of pembrolizumab (Keytruda) 10 mg/kg q3week was undertaken, enrolling three cohorts of patients: MSI-H colorectal cancers, MSI-H non-colorectal cancers, and MSS colorectal cancers [30]. In the initial report from this trial, four (40%) of 10 MSI-H colorectal cancer patients achieved an immune-related objective response with the 20-week immune-related progression free survival (PFS) standing at 78%. Immune-related response assessment relies upon RECIST (Response Evaluation in Solid Tumors) principles, but includes allowance for initial progression in order to account for the uncommon phenomenon of pseudo-progression [36]. Recent updated results have remained encouraging, with 16 (57%) of 25 patients achieving objective responses and an additional nine (32%) with stable disease [30]. Median PFS has not been reached.

Further efforts have been undertaken utilizing the PD-1 inhibitor, nivolumab, in MSI-H tumors. Here, 70 patients with MSI-H tumors were enrolled and treated with nivolumab 3 mg/kg q2week [31]. At the initial presentation, of the 47 patients with at least 12 weeks of follow-up who were evaluable, 12 (26%) achieved an objective response, with a median time to response of 2.12 months (range 1.3–13.6). An additional 14 (30%) of patients achieved stable disease, for a disease control rate of 55%. In a follow-up presentation at the 2017 American Society of Clincial Oncologists (ASCO) GI cancers symposium, objective response rate (ORR) was 31% with 69% of patients achieving stable disease by investigator assessment [37]. While this is less than the pembrolizumab study, these results still suggest significant clinical activity. The reasons for the disparate response rates are not clear and are likely related to differences in the patient populations rather than in differing efficacy of the drugs at hand. Confirmatory studies are underway which utilize PD-1 monotherapy in advanced MSI-H colorectal cancer, including Keynote-164 (NCT02460198).

As in multiple other cancers, the combination of nivolumab and ipilimumab has been evaluated for clinical efficacy. In CheckMate 142, Nivolumab 3 mg/kg q2week was combined with ipilimumab 1 mg/kg q3 week × 4 doses, and followed by nivolumab monotherapy [31]. Thirty patients were initially enrolled, with 27 patients evaluable at the interim analysis. Here, nine (33%) patients achieved objective response, with 14 (52%) achieving stable disease, for a disease control rate of 85%. Because follow-up remains limited and the kinetics of ipilimumab responses can be considerably slower, mature data will be extremely important for evaluation of these two approaches. At this point, the combinatorial approach would seem to offer a higher likelihood of disease control, but only slightly greater probability of response. This comes at the cost of substantially more grade 3/4 immunologic adverse events (AEs). In any case, no firm conclusions can be drawn at present.

With this compelling data, the most recent NCCN guidelines (1.2017) have been modified to include both pembrolizumab and nivolumab as options for metastatic and unresectable MSI-H colorectal cancer. Equally important, in November 2015, pembrolizumab was granted Breakthrough Therapy Designation for MSI-H CRC by the FDA. One would hope and expect that both pembrolizumab and nivolumab will see approval in the months ahead, while data begins to trickle in on various additional approaches.

### 3.2. Microsatellite Unstable (MSI-H) Trials Underway

Multiple studies are ongoing or planned to evaluate the role of PD-1/L1 inhibition in MSI-H colorectal cancer, including the three potentially practice-changing studies described here. A summary of key studies is depicted in Table 2. Keynote-177 is a randomized phase III study which is evaluating patients with Stage IV MSI-H or dMMR colorectal cancer in the first line setting (NCT02563002). In total, 270 patients will be randomized to chemotherapy of the investigators choice vs. Pembrolizumab, at a dose of 200 mg q3 weeks. Crossover will be permitted. The primary endpoint is progression-free survival, with secondary endpoints of overall survival and overall response rate.

The cooperative group system is currently in the process of initiating a similar study, NRG GI004/SWOG1610 (NCT02997228) or COMMITT. This study will utilize a monoclonal antibody which inhibits PD-L1, atezolizumab. In this study, 439 patients with MSI-H/dMMR metastatic colorectal cancer who are treatment naïve will be enrolled and randomized to FOLFOX and bevacizumab, atezolizumab alone, or atezolizumab combined with FOLFOX and bevacizumab. The primary endpoint is progression-free survival. Overall survival, response rates, duration of response, disease control rates and quality of life measures will be among the secondary endpoints.

Another important trial will evaluate the role of PD-L1 inhibition in early Stage MSI-H colon cancer. Also developed through the cooperative group system, Alliance A021502 plans to evaluate FOLFOX ± atezolizumab in 720 patients with Stage III colon cancer (NCT02912559). In the experimental arm, patients will receive 6 months of adjuvant FOLFOX concurrently with atezolizumab, which will then be followed by 6 additional months of atezolizumab monotherapy. The primary endpoint is disease-free survival. Overall survival and adverse events will be the secondary endpoints.

### 3.3. Microsatellite Stable (MSS) Tumors

One informative arm of the pivotal pembrolizumab study enrolled a cohort of patients specifically with MSS colorectal cancer [30]. In this arm, no responses were observed, median PFS stood at 2.2 months and overall survival (OS) at 5 months. These results confirmed the data seen previously in unselected colorectal cancers.

As PD-1 and CTLA-4 have demonstrated synergy in multiple tumor types, recent efforts to explore these combinations have also been pursued in MSS colorectal cancer. From a preliminary report of the Checkmate 142 study, there appears to be only limited efficacy in MSS tumors via this therapeutic strategy [31]. Following use of combined nivolumab and ipilimumab with two different dosing schemes, one (5%) response was observed among 20 patients. Median PFS stood at 2.28 and 1.31 months in the two dosing cohorts. Collectively, this data suggests no generalizable activity of PD-1 monotherapy for MSS colorectal cancer and only very limited activity with PD-1 and CTLA-4 combination therapy. Further details on the one patient who responded could be of great future value in determining whether there are any subsets of MSS tumors where this combination could be considered. An ongoing randomized study will formally evaluate durvalumab (PD-L1 inhibitor) and tremelimumab in the refractory setting (NCT02870920).

### 3.4. MEK and PD-L1 Inhibition

While there is clearly a developing role for PD-1 inhibition in MSI-H colorectal cancer, for MSS colorectal cancer, alternative approaches will be required. CTLA-4 and PD-1/L1 may yet prove beneficial in some subsets of MSS colorectal cancers. However, in the remainder, new drugs and new combinations are sorely needed. Pre-clinical data has suggested multiple opportunities. In melanoma, colorectal, and breast cancer models, MEK inhibition upregulates IFN-gamma mediated HLA molecule and PD-L1 expression [38,39]. In all of these models, MEK inhibition and PD-1 inhibition prove synergistic. Based upon this data, a phase I study of MEK inhibition with cobimetinib and PD-L1 inhibition with atezolizumab (MPDL3280A, Tecentriq) was undertaken with an expansion cohort in KRAS mutant colorectal cancer [35]. In this setting, neither of these drugs would be expected to demonstrate meaningful efficacy as monotherapy. Interim results demonstrated exciting data. Of the 23 mCRC patients enrolled, 20 of whom were KRAS mutant, four (17%) achieved a partial response. A 6-month OS of 72% was achieved, better than might be expected, and on treatment biospecimen suggested immune modulation, similar to the pattern seen pre-clinically. MSI/MMR status was not known for most of the study patients, but of the four responding patients, three were confirmed to be MMR proficient (MSS equivalent). Thus, this has sparked great enthusiasm for expansion of immunotherapy in colorectal cancer. A phase III study is ongoing, where patients with refractory metastatic disease are randomized to cobimetinib and atezolizumab, atezolizumab alone or regorafenib (NCT02788279). Cobimetinib is also being combined with nivolumab and ipilimumab (NCT02060188) as well as being utilized in a trial with atezolizumab and bevacizumab (NCT02876224).

## 4. Additional Immunotherapy Approaches Underway

### 4.1. Combinations with Chemotherapy

Classic cytotoxic therapies are thought to have impact on the tumor microenvironment. It is thought that therapy-induced cell death can be immunogenic, promoting the presentation of tumor antigens which might provoke an adaptive immune response. Both chemotherapy and radiation therapy have been construed to have such properties, though the optimal regimens for induction of immunogenic cell death (ICD) remain to be determined and the true magnitude of this effect remains unclear. Both 5-FU and oxaliplatin have been thought to have a favorable effect [40]. Based on this rationale, FOLFOX is being combined with pembrolizumab in two studies, targeting GI cancers or colon cancer respectively (NCT02375672, NCT02268825).

The potential immune-modulatory role of anti-angiogenic agents was observed in a melanoma study of bevacizumab and ipilimumab. In this study, the blockade of VEGF signaling by bevacizumab combined with ipilimumab increased CD163+ dendritic cell trafficking and and CD8+ T-cell trafficking across the tumor vasculature beyond what was achieved via ipiliumumab alone [41]. When combined, FOLFOX and bevacizumab may decrease granulocytic MDSCs and increase pro-inflammatory helper T-cell (Th17) frequency, rendering a favorable micro-environment for immune checkpoint inhibitor treatment [42].

In a study combining atezolizumab (anti-PD-L1 monoclonal antibody) and bevacizumab with or without chemotherapy (NCT01633970), of the 14 refractory patients who received bevacizumab and atezolizumab, one (7%) experienced response and nine (64%) had stable disease (including two (14%) that were durable for 24 weeks). Another similar study is the phase I trial of the VEGF-TRAP, Aflibercept, and pembrolizumab (NCT02298959).

The combined effects of chemotherapy and anti-angiogenic agents on immune checkpoint therapy are being evaluated clinically. The combination of FOLFOX, bevacizumab and atezolizumab has been investigated in a cohort of 30 patients. In this arm, 23 patients were treatment naïve; in this population, 11 (48%) demonstrated partial response 48% with 20/23 (87%) achieving response or stable disease. Tumor biopsies and peripheral blood demonstrate immune activation [34]. It remains unclear at present whether these results represent any departure from that which would be seen with chemotherapy alone in this population. As it is possible that the response rate does not differ, but durability could be improved, mature data including PFS and OS would be informative.

In a biomarker-driven, multi-arm study, atezolizumab is combined with 5-FU and bevacizumab as maintenance following 8 weeks of FOLFOX and bevacizumab induction (NCT02291289). Separately, the BACCI study is evaluating the impact of adding atezolizumab to Capecitabine and bevacizumab in refractory colorectal cancer (NCT02873195).

### 4.2. Combinations with Radiotherapy

Radiotherapy and thermal therapies have been similarly touted as being immunogenic. For years, rare abscopal responses have been described to radiotherapy, with more recent pre-clinical models suggesting molecules such as PD-1 can otherwise prevent abscopal responses [43]. Multiple planned studies will test the hypothesis that radiotherapy may open a window for successful immunological modulation. To date, few have been completed in colorectal cancer. One of these evaluated a combination of liver directed stereotactic body radiation therapy (SBRT) and the PD-1 inhibitor, AMP224, in patients with metastatic CRC (NCT02298946). Safety was demonstrated; however, no responses were observed [44]. A second study of patients with metastatic CRC has evaluated either radiofrequency ablation or external beam radiotherapy (RT) combined with pembrolizumab (NCT02437071). At the ASCO 2016 Annual Meeting, interim results were made available, revealing one (4.5%) response out of the 22 patients who received one of several doses of short course radiotherapy. The ablation arm had produced no responses to date. Even so, the preclinical data is compelling and multiple additional trials are planned. Dual CTLA-4/PD-1 will be evaluated with various radiotherapy schemas (NCT02888743). Long course chemoradiation is being combined with PD-1 inhibition in locally advanced rectal cancer (NCT02948348, NCT02586610). The value of this approach should become evident in the next few years as these data sets come to surface.

### 4.3. Additional Immunotherapy Combinations

Numerous additional combinations are being studied. Agents that might block suppressive immune factors, such as indoleamine 2,3-dioxygenase (IDO) or LAG-3, are being investigated in phase I trials, combined with PD-1 or PD-L1 inhibitors (NCT02178722, NCT02318277, NCT02327078, NCT02460224). Drugs that are capable of acting as direct immune stimulators, such as KIR and 4-1BB (CD137), are also being studied in multiple combinations, including with PD-1 inhibition (NCT01714739, NCT02179918). There are multiple additional checkpoints and immunomodulatory compounds in development and in phase I investigation. A few of interest are detailed below and a summary is depicted in Table 3.

IDO is an enzyme which breaks down non-dietary tryptophan, potentially having a very important impact in the tumor microenvironment. IDO is upregulated during inflammation by interferons, prostaglandin E2 (PGE2) and multiple other stimuli. In the microenvironment, IDO depletes tryptophan and increases the concentration of breakdown products, kynurenines; the combination potently induces T cell apoptosis. Further, evidence suggests that IDO drives FoxP3+ Treg differentiation, MDSC accumulation and activation [45]. All of these shift the immune milieu to an immunosuppressive, tumor permissive environment. Of relevance, in colorectal cancer patients, IDO has been shown to be associated with lesser CD3+ infiltrating T cells and worse prognosis [46]. Recent phase I studies have been completed with epacadostat (INCB024360), demonstrating reasonable tolerability and successful inhibition of IDO1 activity [47]. Single agent activity appears limited. However, combination data with pembrolizumab suggests high levels of activity in melanoma patients (*n* = 19) with an objective response rate of 58% and complete responses in 26% [48]. This combination, thus far, appears well tolerated as well. Combinatorial data is not yet available for colorectal cancer patients. Naturally, evaluation is being pursued as part of PD-1 inhibitor combination (NCT02327078, NCT02178722), as well as in an ambitious trial that will add epigenetic modulation with azacitidine to the combination, specifically for lung cancer and MSS colorectal cancer (NCT02959437).

Another potentially important immunosuppressive signaling molecule is colony-stimulating factor 1 (CFS1). Macrophage colony-stimulating factor 1 (CSF1R) is a receptor for CSF1, representing a hub which controls differentiation and function of macrophages. Preclinical models have linked the inhibition of CSF1/CSF1R signaling to the reprogramming of the monocytoid population, shifting the population from tumor promoting monocytes (MDSCs) to that of tumor suppressive, antigen presenting macrophages. In these pancreatic cancer models, combinatorial CSF1R and PD-1 or CTLA-4 blockade substantially improved responses [49]. On this basis, a CSF1R inhibitor, Pexidartinib, is being combined with an anti-PD-L1 antibody, durvalumab, in a phase I study with expansion cohorts in colorectal and pancreatic cancer (NCT02777710).

Finally, Cetuximab is a monoclonal antibody which binds to the epidermal growth factor receptor (EGFR) and is approved for use in RAS wt colorectal cancer. As cetuximab is an IgG1 monoclonal antibody, it carries potential to induce antibody dependent cellular cytotoxicity (ADCC). In clinical investigations, cetuximab has been demonstrated to induce an EGFR-specific T-cell response as well as induce antigen spreading in head and neck cancers [50]. In patients with metastatic colorectal cancer who are treated with various chemotherapy combinations, those patients who receive anti-EGFR based therapies demonstrate the most robust intratumoral T-cell infiltrates [51]. Both lines of evidence suggest that cetuximab may favorably alter the tumor immune microenvironment. As such, an ongoing Phase Ib/II investigation is examining the role of cetuximab and pembrolizumab in metastatic colorectal cancer (NCT02713373). Primary endpoints are response rate and 6-month PFS.

## 5. Conclusions

The American Society of Clinical Oncology (ASCO) declared Immunotherapy to be the 2016 Clinical Cancer Advance of the Year. In 2017, the advance of the year has already been announced to be Immunotherapy 2.0. Despite years of frustration, we are beginning to see some success through the use of the immunotherapy approach in colorectal cancer, namely PD-1 inhibition in MSI-H cancers. However, the successful targeting of MSS cancers and non-hypermutated tumors appears to be not too far off on the horizon. MEK and PD-L1 combinations are being rigorously tested, multiple agents and combinations are in development and multiple companies have shifted their focus and investments toward immunotherapeutics. Neglected in this review, but also of note, a case of remarkable success has been witnessed utilizing adoptive cell therapy via tumor infiltrating lymphocytes (TILs) in colorectal cancer [52]. Thus, cancer immunotherapy strategies appear to be moving full speed ahead. Despite the knowledge that many further failures lie in our paths, reason for great optimism remains.

## Figures and Tables

**Table 1:** Key immunotherapy trials in metastatic colorectal cancer (CRC).

Drug(s)	Target	Population	Patients	Response Rate	Identifier
**Trials for MSI-H CRC**					
**Pembrolizumab**	PD-1	Refractory MSI-H CRC	25	57%	Le et al. [30]
**Nivolumab Nivolumab + Ipilimumab**	PD-1	Refractory MSI-H CRC	47	26%	NCT02060188 [31]
	PD-1 + CTLA-4	Refractory MSI-H CRC	30	33%	
**Trials for MSS CRC**					
**Pembrolizumab**	PD-1	Refractory MSS CRC	28	0%	Le et al. [30]
**Nivolumab + Ipilimumab**	PD-1 + CTLA-4	Refractory MSS CRC	20	5%	NCT02060188 [31]
**Trials of Various CRC Sub-Types**					
**Tremelimumab**	CTLA-4	Refractory CRC	49	2%	Chung et al. [28]
**Nivolumab**	PD-1	Refractory CRC	19	0%	Topalian et al. [32]
**BMS-936559**	PD-L1	Refractory CRC	18	0%	Brahmer et al. [33]
**Atezolizumab + Bevacizumab**	PD-L1	Refractory CRC	14	7%	NCT01633970 [34]
**Atezolizumab + FOLFOX/bev**		Metastatic CRC (70% first line)	30	40% (total) 48% (first-line)	
**Atezolizumab + Cobimetinib**	PD-L1 MEK	Refractory CRC (30% MSS, 70% unknown)	23	17% (3 MSS, 1 unknown)	NCT01988896 [35]

**Table 2:** Key ongoing/planned trials in MSI-H CRC.

Patient Population	Treatment	Primary Endpoint	Identifier
Metastatic: Refractory (Cohort A); or ≥1 Prior Therapy (Cohort B)	Pembrolizumab Monotherapy	Objective Response Rate	Keynote 164 NCT02460198
1st Line Metastatic	Pembrolizumab monotherapy vs. Standard of Care Chemotherapy	Progression-Free Survival	Keynote 177 NCT02563002
1st Line Metastatic	Atezolizumab vs. Atezolizumab + FOLFOX + Bevacizumab vs. FOLFOX + Bevacizumab	Progression-Free Survival	NRG-GI004/S1610 NCT02997228
Stage III	Atezolizumab + FOLFOX vs. FOLFOX alone	Disease-Free Survival	Alliance A021502 NCT02912559

**Table 3:** Combinatorial immunotherapy trials in progress.

Drug(s)	PD-1/PD-L1 Partner (Target)	Description	Identifier
**CRC Specific or CRC Expansion Studies**			
**Atezolizumab**	Cobimetinib (MEK), Bevacizumab (VEGF-A)	Phase I—Metastatic CRC	NCT02876224
**Pembrolizumab**	Cetuximab (EGFR)	Phase Ib/II—Pre-treated CRC	NCT02713373
**Atezolizumab**	Capecitabine, Bevacizumab (VEGF-A)	Randomized Phase II Refractory CRC	NCT02873195
**Durvalumab**	Cediranib (VEGFR, c-kit)	Phase I/II—Refractory CRC Expansion	NCT02484404
**Pembrolizumab**	Nintedanib (VEGFR, PDGFR, FGFR)	Phase I/II—CRC	NCT02856425
**Pembrolizumab**	Napabucasin (STAT3)	Phase I/II Refractory CRC	NCT02851004
**Pembrolizumab**	Oral azacitidine (DNMT), Romidepsin (HDAC1/2)	Phase I—Pre-treated MSS CRC	NCT02512172
**Pembrolizumab**	Azacitidine (DNMT), Epacadostat (IDO-1)	Phase I/II Refractory MSS CRC and NSCLC	NCT02959437
**Nivolumab**	Epacadostat (IDO-1)	Phase I/II—Solid tumors, CRC	NCT02327078
**Pembrolizumab**	Poly-ICLC (TLR-3)	Phase I/II—MSS CRC	NCT02834052
**Nivolumab**	Varlilumab (CD-27)	Phase I/II—Solid tumors, CRC	NCT02335918
**Durvalumab**	Pexidartinib (CSF-1R)	Phase I—Pre-treated pancreas and CRC	NCT02777710
**Atezolizumab**	CPI-444 (Adenosine-A2A)	Phase I—Solid tumors, MSI-H CRC	NCT02655822
**Nivolumab**	Chemoradiation	Phase I/II—Locally advanced rectal cancer	NCT02948348
**Durvalumab**	Tremelimumab (CTLA-4), Radiation	Phase II—NSCLC and CRC with liver metastases	NCT02888743
**Pembrolizumab**	Tumor infiltrating Lymphocytes, IL-2, cytoxan, fludarabine	Phase II—digestive tumors, CRC arm	NCT01174121
**Phase I Studies in Solid Tumors**			
**Durvalumab**	Selumetinib (MEK)	Phase I—Solid Tumors	NCT02586987
**Pembrolizumab**	Aflibercept (VEGF-A/B, PIGF)	Phase I—solid tumors	NCT02298959
