# Erratum: Boland, P.M., et al. Immunotherapy for Colorectal Cancer, *Cancers* 2017, *9*, 50

**DOI:** 10.3390/cancers12051320

**Published:** 2020-05-22

**Authors:** Patrick M. Boland, Wen Wee Ma

**Affiliations:** 1 Roswell Park Cancer Institute, Department of Medicine, Elm and Carlton St, Buffalo, NY 14263, USA; Patrick.Boland@RoswellPark.org; 2 Mayo Clinic, Division of Medical Oncology, 200 First St. SW, Rochester, MN 55905, USA

The authors wish to make the following corrections to this paper [1]:

The funding information was missing in the original version. The following acknowledgement of funding support should be included: 

**Funding:** This work was partially supported by American Cancer Society Internal Research Grant #126771-IRG-14-194-11-IRG.

These changes do not affect the scientific results. The manuscript will be updated and the original version will remain online on the article webpage, with a reference to this erratum. The authors would like to apologize for any inconvenience caused to the readers by this change.
